# Deletion or Inhibition of NOD1 Favors Plaque Stability and Attenuates Atherothrombosis in Advanced Atherogenesis

**DOI:** 10.3390/cells9092067

**Published:** 2020-09-10

**Authors:** Silvia González-Ramos, Victoria Fernández-García, Miriam Recalde, Cristina Rodríguez, José Martínez-González, Vicente Andrés, Paloma Martín-Sanz, Lisardo Boscá

**Affiliations:** 1 Instituto de Investigaciones Biomédicas Alberto Sols (CSIC-UAM), 28029 Madrid, Spain; bvfernandez@iib.uam.es (V.F.-G.); miriamrecal@hotmail.com (M.R.); pmartins@iib.uam.es (P.M.-S.); 2 Centro de Investigación Biomédica en Red en Enfermedades Cardiovasculares (CIBERCV), 28029 Madrid, Spain; crodriguezs@santpau.cat (C.R.); jose.martinez@iibb.csic.es (J.M.-G.); vandres@cnic.es (V.A.); 3 Institut de Recerca del Hospital de la Santa Creu i Sant Pau-Programa ICCC, IIB Sant Pau, 08041 Barcelona, Spain; 4 Instituto de Investigaciones Biomédicas de Barcelona (IIBB-CSIC), IIB Sant Pau, 08041 Barcelona, Spain; 5 Laboratory of Molecular and Genetic Cardiovascular Pathophysiology, Vascular Pathophysiology Area, Centro Nacional de Investigaciones Cardiovasculares Carlos III (CNIC), 28029 Madrid, Spain; 6 Centro de Investigación Biomédica en Red de Enfermedades Hepáticas y Digestivas (CIBERehd), 28029 Madrid, Spain

**Keywords:** coronary disease, atherothrombosis, vulnerable plaque, innate immunity, pattern recognition receptors

## Abstract

Atherothrombosis, the main cause of acute coronary syndromes (ACS), is characterized by the rupture of the atherosclerotic plaque followed by the formation of thrombi. Fatal plaque rupture sites show large necrotic cores combined with high levels of inflammation and thin layers of collagen. Plaque necrosis due to the death of macrophages and smooth muscle cells (SMCs) remains critical in the process. To determine the contribution of the innate immunity receptor NOD1 to the stability of atherosclerotic plaque, *Apoe^−/−^* and *Apoe^−/−^ Nod1^−/−^* atherosclerosis prone mice were placed on a high-fat diet for 16 weeks to assess post-mortem advanced atherosclerosis in the aortic sinus. The proliferation and apoptosis activity were analyzed, as well as the foam cell formation capacity in these lesions and in primary cultures of macrophages and vascular SMCs obtained from both groups of mice. Our results reinforce the preeminent role for NOD1 in human atherosclerosis. Advanced plaque analysis in the *Apoe^−/−^* atherosclerosis model suggests that NOD1 deficiency may decrease the risk of atherothrombosis by decreasing leukocyte infiltration and reducing macrophage apoptosis. Furthermore, *Nod1^−/−^* SMCs exhibit higher proliferation rates and decreased apoptotic activity, contributing to thicker fibrous caps with reduced content of pro-thrombotic collagen. These findings demonstrate a direct link between NOD1 and plaque vulnerability through effects on both macrophages and SMCs, suggesting promising insights for early detection of biomarkers for treating patients before ACS occurs.

## 1. Introduction

According to the World Health Organization, acute coronary syndromes (ACS) are the leading cause of morbidity and mortality in Western society. Rupture of the atheroma underlies most of the thrombotic events that manifest clinically as coronary artery disease, stroke, transient ischemic attack, and peripheral arterial disease [1,2,3]. Acute coronary events mediated by thrombi depend mainly on the composition and vulnerability of the plaque. The trapping of cholesterol-loaded foam cells [4] causes thinning of the fibrous cap (FC) and expansion of the necrotic core (NC), as a result of the combination of vascular smooth muscle cells (SMC) and macrophages proliferation and excessive cell death [5,6]. In combination with degradation of the extracellular matrix by proteases released from apoptotic foam cells, the FC is destabilized, leading to its breakdown or erosion, and subsequent exposure to the bloodstream of NC content. As a result, platelets and fibrin aggregate to form a thrombus resulting in partial or total ischemic blockage of the artery [7,8]. Therefore, the clinical atherosclerotic lesion is essentially an unresolved inflammatory condition leading to a vulnerable plaque [9,10]. Notably, atherosclerosis is strongly associated with systemic risk factors (e.g., high LDL, infections, diabetes) where the cellular components of the innate immune system are relevant. In this regard, the nucleotide-binding oligomerization domain (NOD)-1 receptor of the innate immune system appears to play a key role. Our group has recently identified endothelial NOD1 as proatherogenic in response to oxLDL and a conserved region of bacterial peptidoglycan iE-DAP (γ-D-glutamyl-meso-diaminopimelic acid) [9]. The downstream interaction of NOD1 with receptor-interacting protein-2 (RIP2 or RICK) activates the canonical nuclear factor kappa B (NF-κB) signaling pathway [11] that upregulates the expression of adhesion molecules of endothelial cells and the concomitant recruitment of monocytes and neutrophils to the vasculature.

Although interest in the study of NOD1 in cardiovascular diseases has increased lately, [12,13,14,15] its role in destabilizing atherosclerotic plaque is still lacking. In this study, our aim is to investigate the contribution of NOD1 to key characteristics of plaque vulnerability. We show that NOD1 is induced in SMC and macrophages in human atherosclerotic tissues. Furthermore, the inactivation of *Nod1* in the *Apoe^−/−^* mouse model of atherosclerosis may contribute to plaque stability by modulating the pathophysiological functions of macrophages and SMCs.

## 2. Materials and Methods

### 2.1. Human Samples

Human coronary arteries were collected from patients undergoing heart transplant at the Hospital de la Santa Creu i Sant Pau (Barcelona, Spain). Atherosclerotic and non-atherosclerotic coronary arteries were taken from coronary artery disease (CAD) and non-CAD patients, respectively. Written consent was obtained from all participating subjects. The studies were approved by the Ethics Committee of the hospital and were conducted in accordance with the Helsinki Declaration (project RTI2018-094727-B-100; approved October 2018).

### 2.2. Animal Procedures

Animal studies were approved by the local ethics committee, and all animal procedures conformed to EU Directive 2010/63 and Recommendation 2007/526/EC regarding the protection of animals used for experimental and other scientific purposes, enforced in Spanish law under Real Decreto 53/2013. C57BL/6 (*Wt*) and *Apoe^−/−^* mice were obtained from Charles River (JAX mice stock #000664 and #002052, respectively. Barcelona, Spain). Double-knockout *Apoe^−/−^Nod1^−/−^* mice were generated by crossing *Apoe^−/−^* mice with *Nod1^−/−^* mice as previously described [9]. All experiments compared male *Apoe^−/−^* mice vs. male *Apoe^−/−^Nod1^−/−^* littermates. In order to accelerate the development of atherosclerotic lesions, at 8 weeks of age, males were placed on high-fat diet (HFD, 10.2% hydrogenated coconut oil, 0.75% cholesterol; Ssniff, Soest, Germany) for 16 weeks. Mice were anaesthetized intraperitoneally under general anesthesia (ketamine/xylazine combination at 80 mg/kg and 10 mg/kg body weight, respectively) before euthanasia by CO_2_ inhalation.

Whole blood was extracted post-mortem by cardiac puncture and plasma was obtained by centrifugation at 2000× *g* for 10 min at 4 °C. Plasma concentrations of total cholesterol, free cholesterol, LDL-cholesterol, HDL-cholesterol, and triglycerides were measured enzymatically using kinetic colorimetric kits (Spinreact, St Esteve de Bas, Girona, Spain) according to manufacturer’s instructions.

### 2.3. Cell Procedures

Smooth muscle cells (SMC) were harvested from abdominal and thoracic aortas from 2-month-old animals as previously described [16] and cultured in Dulbecco’s modified Eagle medium (DMEM, GIBCO, Madrid, Spain) containing 20% fetal bovine serum (FBS, Lonza, Barcelona, Spain), L-glutamine and antibiotics (100 units/mL penicillin and 100 µg/mL streptomycin). Bone marrow-derived macrophages (BMDM) were obtained from femoral bone marrow suspensions [9] differentiated for 7 days in the presence of DMEM plus 10% FBS and 20 ng/mL macrophage colony-stimulating factor (M-CSF, PeproTech, London, UK).

For in vitro apoptosis studies, cells were either irradiated with ultraviolet (UV) light (BMDM: 80 J/m^2^, SMC: 120 J/m^2^) and cultured an additional 24 h (BMDM) or 48 h (SMC) period, or incubated with the nitric oxide donor *S*-nitrosoglutathione (GSNO, 1 mM, Sigma, Madrid, Spain); with oxidized LDL (oxLDL, 50 µg/mL, Biochemistry-Research Unit at Instituto Ramón y Cajal de Investigación Sanitaria, Madrid, Spain); with the NOD1 agonist c12-iE-DAP (1 µg/mL, Invivogen, San Diego, CA, USA) or with inactive analogue iE-Lys, for 24 h in BMDM cultures or 48 h in SMC cultures. For apoptosis analysis by flow cytometry, control and treated cells were collected and labelled with propidium iodide as per manufacturer’s instructions (ThermoFischer Sci., Madrid, Spain) or processed for cleaved caspase-3 staining as follows: cells were fixed with 1% paraformaldehyde, permeabilized with 0.1% Triton-X100, and stained with a rabbit polyclonal anti-cleaved caspase 3 antibody (1/600, Cell Signaling Technology, Danvers, MA, USA), followed by Alexa Fluor 647-conjugated goat anti-rabbit IgG secondary antibody (1/500, Invitrogen, Carlsbad, CA, USA). Nuclei were counterstained with DAPI (Life Technologies). Flow cytometry was conducted in a FACSCanto II and DNA histograms were fitted into cell-cycle distributions using the BD FACSDiva software (Beckton Dickinson, Madrid, Spain). Apoptotic cells were identified as the sub-G0 peak subpopulation after propidium iodide staining or as cleaved caspase-3-positive cells.

For SMC, FACS-based cell-cycle analysis, cells were synchronized in G0/G1 by 72 h serum deprivation (DMEM plus 0.1% FBS) and then restimulated with DMEM supplemented with 20% FBS for 12 h and 24 h. Cells were labelled with propidium iodide as described earlier, and processed to fit into same cell-cycle distributions as for FACS-based apoptosis analysis.

For NOD1 signaling cascade analysis in SMC or BMDM, cells were pre-incubated when indicated with the NOD1 antagonist Nodinitib-1 (1 µM, Cayman, Madrid, Spain) for one hour and then treated with LDL (50 or 30 µg/mL), oxidized LDL (20 or 50 µg/mL), iE-Lys (a structural analogue of c12-iE-DAP that does not activate NOD1) or the NOD1 activator c12-iE-DAP (1 µg/mL, Invivogen) for 24 h.

To detect foam cell formation, BMDM and SMC were incubated with 50 µg/mL LDL or oxLDL for 24 h and 48 h, respectively, fixed in 1% paraformaldehyde and stained with 0.5% Oil red O (ORO, Sigma) in isopropanol and counterstained with hematoxylin.

### 2.4. Histological Analysis and Lesion Quantification

After mouse cardiac perfusion with PBS supplemented with 5 mM of EDTA, mouse hearts were harvested and fixed in 4% paraformaldehyde (PFA) for 24 h at 4 °C, incubated 24 h in PBS supplemented with 30% sucrose, embedded in OCT and cryopreserved at −70 °C.

Cryocut cross-sections (5 µm) of aortic roots were evaluated for conventional hematoxylin-eosin (HE) staining, 0.1% sirius red to detect collagen or 0.5% ORO to detect neutral lipids. Images were captured with a Zeiss Axiophot microscope with a Plan-NEOFLUAR 10x/0.3 objective (Zeiss, Oberkochen, Germany) and a DP70 camera (Olympus, Madrid, Spain). Polarized images were obtained using a Mirax digital slide scanner (3DHistech, Budapest, Hungary). To avoid specific biases due to potential differences in lesion shape, cross sections of the entire lesion were analyzed and averaged [17].

To obtain the aortas for the analysis, after fixing in PFA overnight at 4 °C, the aortas were whole-mount stained with 0.2% ORO in methanol, opened longitudinally and pinned to black wax to expose the entire luminal surface. Images were acquired using a Leica MZ6 SZX10 stereomicroscope (Leica Microsystems, Wetzlar, Germany) coupled to a Leica DFC300 digital color camera (Leica Microsystems). The planimetric area of atherosclerotic plaques was measured in pixels using ImageJ.

### 2.5. Immunostaining

Human arteries were fixed overnight in 4% PFA/0.1 M PBS (pH 7.4), embedded in paraffin and sectioned into 5 μm sections with a microtome (Jung RM2055, Leica). Consecutive deparaffinized sections were rehydrated, subjected to antigen retrieval in 10 mM citrate buffer (pH 6.0), blocked and incubated with a rabbit polyclonal antibody against NOD1 (1:40, Abcam, Cambridge, UK), with a mouse monoclonal anti-smooth muscle α-actin (SMA) alkaline phosphatase-conjugated antibody (1:200, Sigma) or with a rat monoclonal antibody against anti-Mac3 (1:100, Santa Cruz, Santa Cruz, CA, USA). After extensive washes, sections were incubated with correspondingly biotinylated goat anti-rabbit or goat anti-rat secondary antibodies (Vector). Immunocomplexes were detected after incubation with Vectastain Elite ABC reagent (PK6100, Vector, Barcelona, Spain) and DAB substrate (Roche). Images were acquired with an Olympus Vanox AHBT3 microscope and digitalized by a Sony camera (DXC-S500).

For immunostaining of cryo-section samples, slides were stained with antibodies specific for mouse Mac3 (1:200, Becton Dickinson), Ly6g (1:100, Becton Dickinson), cleaved caspase 3 (1/600, Cell Signaling Technology) and Ki-67 (1:200, Abcam), followed by secondary staining using standard procedures. Secondary antibodies for immunofluorescence were Alexa Fluor 647-conjugated anti-rabbit (Invitrogen) and Alexa Fluor 594-conjugated anti-rat (Invitrogen). SMC were identified with mouse anti-smooth muscle α-actin FITC-conjugated antibody (1:1000, Sigma). Nuclei were counterstained with DAPI (Life Technologies, Madrid, Spain). Immunofluorescence staining of cryo-sections were mounted in Prolong Gold Antifade mounting medium (Life Technologies). Primary control panel was performed with an appropriate isotype control IgG and secondary controls incubations were performed in the absence of primary antibody.

A LSM710 confocal microscope with a Plan-APOCHROMAT 25x/0.8 oil immersion objective (Zeiss) was used to capture images from immunofluorescence staining. Images were analyzed using ImageJ and were processed for presentation with Zen2009 software.

### 2.6. Western Blot Analysis

After stimulation of primary cultures, cells were washed twice with ice-cold PBS. Whole protein extracts were obtained using ice-cold proprietary detergent in 25 mM bicine, 150 mM NaCl; pH 7.6 (T-PER^®^ Tissue Protein Extraction Reagent, Thermo Fisher Sci.) supplemented with phosphatase cocktail and protease inhibitors (Sigma) [5,18].

Proteins were resolved on SDS-PAGE gels and then transferred to nitrocellulose membranes. Proteins were detected using rabbit polyclonal antibody against NOD1 (1:500, Abcam), rabbit polyclonal antibody against phospho-RIP2 (1:1000, Cell Signaling), rabbit polyclonal antibody against RIP2 (1:1000, Cell Signaling), rabbit polyclonal antibody against phospho-NF-κB p65 (1:3000, Santa Cruz), mouse monoclonal antibody against NF-κB p65 (1:1000, Santa Cruz), mouse monoclonal antibody against β-actin (1: 40,000, Sigma), and HRP-conjugated secondary antibodies (BioRad, Hercules, CA, USA).

Protein bands were visualized using a Luminata chemiluminescence detection system (Merck Millipore, Madrid, Spain) and a ImageQuant LAS 500 imager (GE Healthcare Life Sci., Madrid, Spain) and were quantified using ImageJ (National Institutes of Health). Protein band intensities of interest were expressed as a percentage of those of the β-actin bands as indicated.

### 2.7. qRT-PCR

Total RNA was isolated by homogenization in QUIAZOL^®^ by a TissueLyser LT and eluted using MinElute columns (Qiagen; Madrid, Spain). RNA integrity was assessed by RNA Nano Chip (Agilent Technologies; Madrid, Spain). 250 ng of RNA were retro-transcribed by using High-Capacity cDNA Reverse Transcription Kit (Applied Biosystems; Madrid, Spain). SYBR Green assay was conducted in 7900HT Fast Real-Time PCR System equipment for qRT-PCR detection of *Cd36* (5′-AGATGACGTGGCAAAGAACAG-3′ and 5′-CCTTGGCTAGATAACGAACTCTG-3′), *Sr-a* (5′-GAGCCTCGTTCACAGGAGTC-3′ and 5′-CACCAGCTCTAGCATGTCCTC-3′) and *Rplp0* (5′-ACTGGTCTAGGACCCGAGAAG-3′ and 5′-TCCCACCTTGTCTCCAGTCT-3′).

Calculations were made from measurement of technical triplicates of each sample. The relative amount of mRNA was calculated with the comparative 2-ΔΔCt method using mouse *18S* or *36b4* as endogenous control transcripts.

### 2.8. Quantification and Statistical Analysis

All values are expressed as means ± s.e.m. Statistical calculations were performed using GraphPad Prism 6 (GraphPad Software Inc.; San Diego, CA, USA). After calculating for normality by D’Agostino–Pearson omnibus test, either a non-parametric test (Mann–Whitney U-test), or a normality test (unpaired Student’s t test with Welch’s correction) was used as appropriate. Statistical significance was deemed at *p* values < 0.05. Removal of outliers was assessed by ROUT method. Statistical tests and *p* values are specified for each panel in the respective figure legends. *n* indicated in the figure legends refers to the number of individual animals for in vivo and ex vivo assays.

## 3. Results

### 3.1. NOD1 in Vascular Smooth Muscle Cells and Macrophages Plays a Key Role in Murine and Human Atherosclerosis Plaque Formation

**Figure 1:**
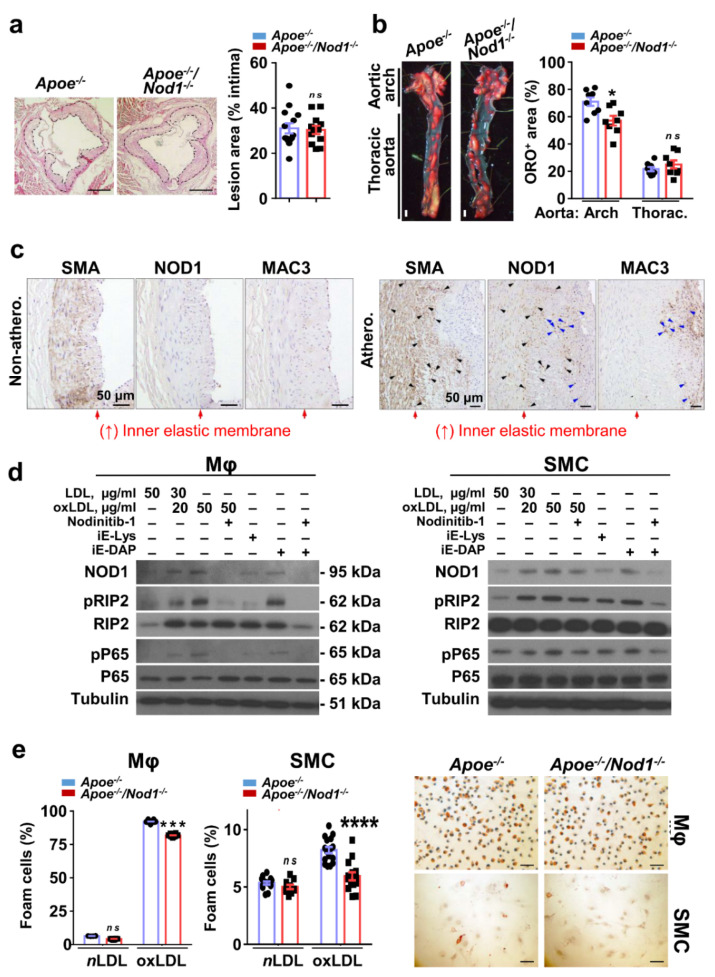
Activation of nucleotide-binding oligomerization domain (NOD)-1 signaling pathway in macrophages and smooth muscle cells (SMC) in advanced atherosclerosis. (**a**) Quantification of lesion area in the semilunar valve cusps of *Apoe^−/−^* (*n* = 14) and *Apoe^−/−^Nod1^−/−^* (*n* = 12) mice fed high-fat diet (HFD) for 16 weeks. (**b**) Quantification of positive Oil Red O (ORO) lesion area in the indicated aortic regions in the same cohort. Panels show representative en face ORO staining of aortas from these animals. (**c**) Representative images for co-localization of smooth muscle α-actin (SMA), NOD1 and Mac3 immunohistochemistry in the arterial intimal thickening of non-atherosclerotic (non-athero.) and atherosclerotic (athero.) human coronary arteries. Arrows point out NOD1^+^ cells of macrophages (blue) and smooth muscle cells (black) in the lesion area. Red arrows delimit internal elastic lamina. (**d**) Immunoblot analysis and representative panel of NOD1, *p*RIP2, RIP2, pP65, and P65 in *Wt* BMDM and SMC pre-treated with the NOD1 inhibitor Nodinitib-1 and/or stimulated with native LDL (as control for lipid load in the medium), oxLDL, iE-Lys (an inactive NOD1 activator) and c12-iE-DAP (an agonist for NOD1) for 24 h or 48 h, respectively. Protein levels were normalized to tubulin. (**e**) *Apoe^−/−^* and *Apoe^−/−^Nod1^−/−^* macrophages (Mφ) and SMC were exposed to native LDL or oxLDL for 24 h or 48 h respectively, and then ORO stained. Representative images for oxLDL treatment and quantification are shown of stained cells in three independent experiments. Data are represented as mean ± s.e.m. of the indicated number (*n*) of repeats. * *p* < 0.05, *** *p* < 0.001 **** *p* < 0.0001 vs. *Apoe^−/−^* by Student’s *t* test. Bars, 100 μm (**a**), 1 mm (**b**), 50 µm (**c**,**e**).

To investigate the role of NOD1 in the advanced stages of atherosclerosis, *Apoe^−/−^Nod1^−/−^* mice and *Apoe^−/−^* controls were placed in a HFD for 16 weeks. Although no significant differences were found between the groups in the lesion area of the aortic valve (Figure 1a), the planimetric analysis of the aorta stained with ORO showed an approximate reduction of 15% in the atheroma of the aortic arch in *Apoe^−/−^Nod1^−/−^* mice compared to controls (Figure 1b), thus confirming our previous results on the preventive role of *Nod1* deletion in early atherosclerosis [9]. Notably, the body weights of *Apoe^−/−^Nod1^−/−^* and *Apoe^−/−^* mice remained similar after 16 weeks with HFD (Figure S1a). Likewise, the plasma lipid concentrations after the HFD regimen did not show statistical differences when comparing *Apoe^−/−^Nod1^−/−^* and *Apoe^−/−^* groups (Figure S1b).

When we evaluated the expression of SMA, NOD1, and MAC3 in consecutive sections of human atherosclerotic coronary arteries, we observed a marked NOD1 staining in cells near the lipid deposition areas, while the staining was much weaker in the non-atherosclerotic coronary controls. In fact, immunohistochemical analysis of human plaques of SMC and macrophages markers revealed elevated expression of NOD1 in both cell types (Figure 1c and Figure S2). These results, together with our previous studies [9] suggest a critical role for NOD1 in the key cell players of human and mice atherosclerosis. The accumulation of oxLDLs in the intima is crucial in the plaque lifetime. Given the fact that these oxidized lipids tightly regulate *Nod1* expression, we next determined whether they could also regulate its expression in the two main cell components of the plaque. Treatment of BMDM and SMC with oxLDL, but not with LDL (used as controls for lipid composition of the culture medium), activated the NOD1 signaling cascade in both cell types, an effect suppressed after NOD1 inhibition by Nodinitib-1 (Figure 1d and Figure S3 for quantification of the blots). Moreover, NOD1 activation with the agonist iE-DAP, but not the inactive analogue iE-Lys, promoted RIP-2 and p65 phosphorylation, an effect suppressed when macrophages were treated with Nodinitib-1 (Figure 1d)**.** The effects on SMC were less intense, but inhibition of NOD1 by Nodinitib-1 confirmed the relevance of this innate immune receptor in RIP-2 signaling in these cells (Figure 1d). SMC proliferation and macrophage foam cell formation are especially important hallmarks in advanced atherosclerosis lesions [4,19]. Therefore, we analyzed in vitro foam cell development in both cell types in response to oxLDL. ORO staining of intracellular lipids indicated that while macrophages and SMC isolated from *Apoe^−/−^Nod1^−/−^* and *Apoe^−/−^* mice engulfed similarly unmodified LDL, fewer *Apoe^−/−^Nod1^−/−^* macrophages and SMC engulfed oxLDL (Figure 1e). Analysis of the scavenger receptor *CD36, Olr1* (*Lox1*, oxidized low-density lipoprotein receptor 1) and *Cd68* (LDL scavenger receptor) mRNA in the aortic arch of HFD fed *Apoe^−/−^* and *Apoe^−/−^Nod1^−/−^* did not show differences between both groups; however, a modest increase in *Msr1* (*Sr-a*; macrophage scavenger receptor 1) mRNA, but statistically significant, was measured, indicating that lipid uptake via these receptors was not reduced by *Nod1* deficiency [20,21,22] (Figure S4). The analysis of total lipid in the aortic valve of the same mice (Figure S5) did not reveal a biological significance for NOD1 in foam-cell formation in advanced atherosclerosis.

### 3.2. NOD1 Deficiency Modulates Structural and Compositional Features of Vulnerable Plaques

**Figure 2:**
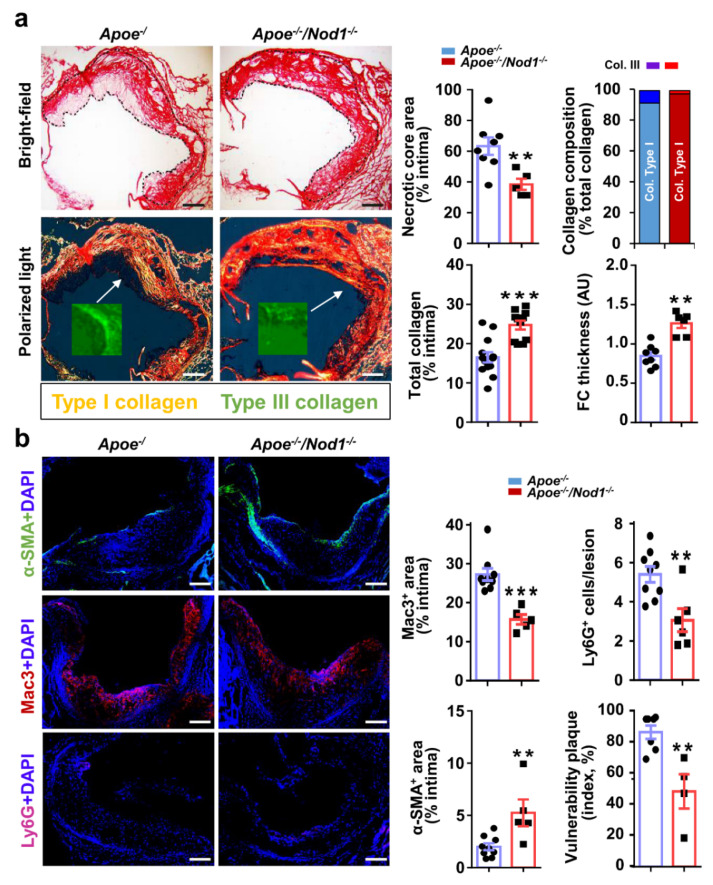
*Nod1* deficiency in *Apoe^−/−^* mice results in decreased vulnerable plaques. (**a**) The collagen content of aortic sinus plaques in *Apoe^−/−^* (*n* = 13) and *Apoe^−/−^Nod1^−/−^* (*n* = 11) mice fed HFD for 16 weeks was evaluated by Sirius red staining. Quantification of total collagen content, necrotic core (NC) area and fibrous cap (FC) thickness was analyzed using brightfield microscopy. Hughe birefringence under polarized light illumination allowed quantification of type I (‘mature-collagen’; orange red birefringence) or type III (‘immature-collagen’; green yellow birefringence and green image in the inset) collagen as percent of total collagen. Representative images in bright field and polarized light illumination in lesions of the aortic sinus are shown. (**b**) Quantification of macrophage, neutrophil and SMC neointimal content in the semilunar valve cusps of the same cohort of mice. Vulnerability plaque index was determined as the NC and MAC3^+^ areas divided by the collagen and smooth muscle α-actin (α-SMA)+ staining areas of the same lesions [24]. Representative immunofluorescence images of anti-MAC3, anti-Ly6G and anti-α-SMA staining in lesions of the aortic sinus are shown. Data are represented as mean ± s.e.m. of the indicated number (*n*) of repeats. ** *p* < 0.01, *** *p* < 0.001 vs. *Apoe^−/−^* by Student’s *t* test. Bars, 50 μm.

We also examined the consequences of inactivating *Nod1* on plaque composition. Human vulnerable plaques are typically associated with the presence of a highly inflammatory cell content and a large NC covered by a thin FC, the latter being characterized by decreased content of SMC and collagen [23]. The extent of the NC in relation to the size of the plaque was greater in *Apoe^−/−^* than in *Apoe^−/−^Nod1^−/−^* mice, while the FC was thicker in *Apoe^−/−^Nod1^−/−^* mice (Figure 2a). Consistent with these results, collagen evaluation by picosirius red staining showed a higher percentage of total positive area within the intima in *Apoe^−/−^Nod1^−/−^* than in *Apoe^−/−^* mice. Further analysis of collagen composition revealed a significant difference between type I and type III collagen, demonstrating a higher proportion of thick fibers in *Apoe^−/−^Nod1^−/−^* mice than in *Apoe^−/−^* mice (Figure 2a, inset). Therefore, although *Apoe^−/−^* mice are not particularly prone to develop unstable plaques [24], our analysis points towards the idea that *Nod1* deficiency might favor plaque-stabilizing factors.

To further assess the progression of atheromas in *Apoe^−/−^Nod1^−/−^* and *Apoe^−/−^* mice, we determined the inflammatory and the SMC content in the aortic cusps, the region containing the most advanced lesions in the *Apoe*-deficient model [25]. Analysis of the aortic root revealed a lower content of intimal neutrophils and macrophages in *Apoe^−/−^Nod1^−/−^* lesions compared to *Apoe^−/−^* mice. Not only this, but the intimal positive area for smooth-muscle actin was significantly higher in *Apoe^−/−^Nod1^−/−^* than in *Apoe^−/−^* mice, compatible with a lower overall vulnerability in *Apoe^−/−^Nod1^−/−^* atherosclerotic lesions (Figure 2b). These results are consistent with those obtained when analyzing the leukocyte content in the blood and in the spleen (Figures S6 and S7).

### 3.3. Nod1 Inactivation Increases SMC Proliferation and Reduces Macrophage Apoptosis

**Figure 3:**
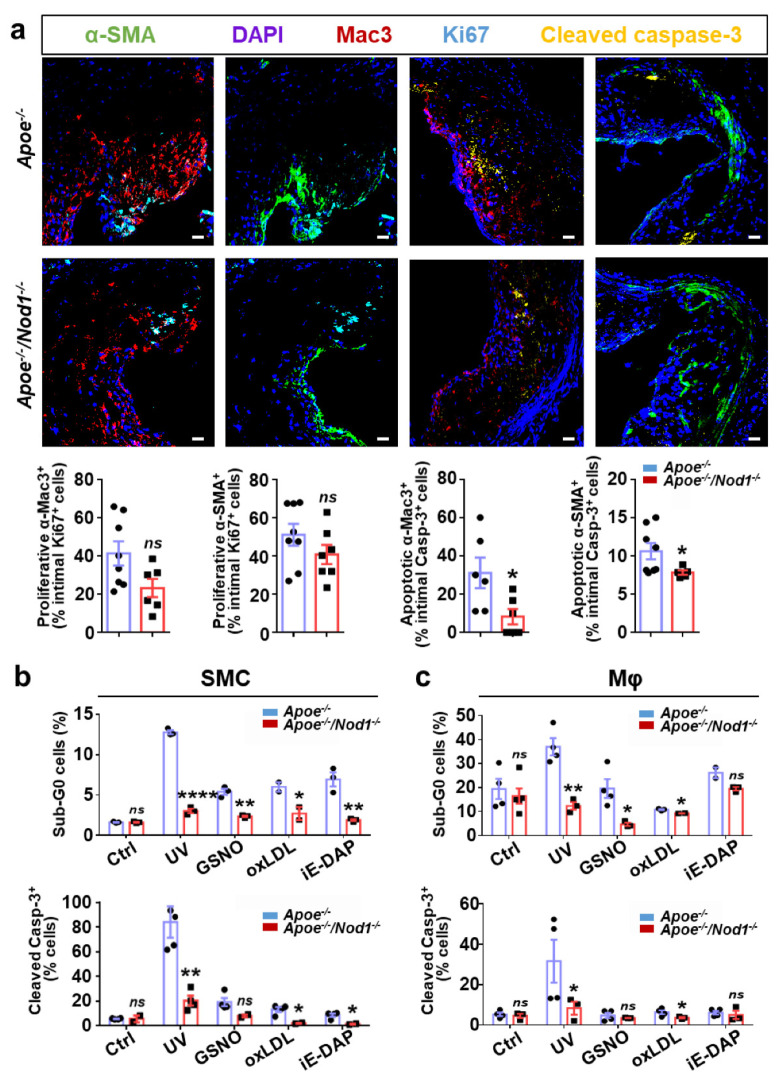
Lack of *Nod1* reduces apoptotic activity of SMC and macrophages. (**a**) Cross-sections from the aortic sinus of *Apoe^−/−^* (*n* = 6) and *Apoe^−/−^Nod1^−/−^* (*n* = 6) mice fed HFD for 16 weeks were doubly stained with Ki-67/MAC-3, cleaved caspase-3/MAC-3 (top) or Ki-67/smooth muscle α-actin (α-SMA), cleaved caspase-3/α-SMA (bottom) to appropriately identify not only proliferative macrophages or in apoptosis (top), but also SMC (bottom) in proliferative or apoptotic state. The results for proliferation are presented as percentage of cells doubly positive for Ki-67 and MAC-3 or α-SMA relative to total number of Ki-67^+^ cells within the atheroma. The results for apoptosis are presented as percentage of cells doubly positive for cleaved caspase-3 and MAC-3 or α-SMA relative to total number of cleaved-caspase-3^+^ cells within the atheroma. SMC (**b**) and macrophage (Mφ) (**c**) apoptotic cells were identified as the sub-G0 population after propidium iodide staining by flow cytometry (upper panels) or as cleaved caspase-3 immunoreactive cells by confocal microscopy (lower panels). Cells were either untreated, irradiated with ultraviolet (UV) light (80 J/m^2^ and harvested after 24 h for BMDM; 120 J/m^2^ and harvested after 48 h for SMC), or incubated with 1 mM S-nitrosoglutathione (GSNO), 50 µg/mL oxidized LDL (oxLDL) or 1 µg/mL c12-iE-DAP for 24 h (BMDM) or 48 h (SMC). Results using both methods represent the average of three independent experiments. Data are represented as mean ± s.e.m. of the indicated number (*n*) of repeats. *p < 0.05, **p < 0.01, ****p < 0.0001 vs. *Apoe^−/−^* by Mann–Whitney U test (**a**) or by Student’s *t* test (**b**,**c**). Bars, 20 μm.

We next seek to investigate the mechanisms underlying the atheroprotective action of the *Nod1* deletion in both SMC and macrophages. Cell proliferation and apoptosis play equally important roles in atherosclerosis [26]. Interestingly, *Apoe^−/−^Nod1^−/−^* SMC entered earlier into the S phase of the cell cycle compared to *Apoe^−/−^* controls, as determined by flow cytometry analysis of starvation-synchronized cultures (Figure S8). These studies demonstrated a significantly higher percentage of cells in G_1_/G_0_ and S-phases in *Apoe^−/−^* vs. *Apoe^−/−^Nod1^−/−^* SMC at all-time points analyzed (0, 12, and 24 h). Moreover, the differences in the percentages of *Apoe^−/−^* cell cultures in S-phase reached statistical significance after 24 h of serum restimulation compared to 0 h (*p*-value < 0.01), while in *Apoe^−/−^Nod1^−/−^*, differences in S-phase reached statistical significance 12 h after serum restimulation (*p*-value < 0.05). Taken together, these findings suggest that serum-restimulated *Apoe^−/−^Nod1^−/−^* SMC re-enter the cell cycle faster than *Apoe^−/−^* controls. Despite the increased proliferative capacity of cultured SMC lacking *Nod1*, cell proliferation was similar in cross-sections of the aortic root from *Apoe^−/−^Nod1^−/−^* and *Apoe^−/−^* mice fed HFD for 16 weeks (Figure 3a), as estimated by the neointimal content of vascular smooth muscle and macrophages positives for Ki67.

To assess the role for NOD1 in plaque’s apoptosis, aortic root cross-sections were examined using double-staining experiments to identify apoptotic neointimal macrophages (cleaved caspase-3/Mac3) and SMC (cleaved caspase-3/SMA). We found a significant reduction of both apoptotic cell types in *Apoe^−/−^Nod1^−/−^* atheromas vs. the corresponding *Apoe^−/−^* lesions (Figure 3a; the results are expressed as percentage of cells doubly positive for cleaved caspase-3 and Mac3, or SMA relative to the total number of cleaved caspase-3-positive cells within the atheroma). Not only this, but while in *Apoe^−/−^Nod1^−/−^* intima the number of apoptotic macrophages and SMC was strictly similar, its content in *Apoe^−/−^* mice was 3-fold in favor of apoptotic macrophages vs. SMC, highlighting that the *Nod1* deletion has a strong impact on macrophage apoptosis.

To further investigate the role of NOD1 in controlling macrophage and SMC apoptosis, we prepared primary cultures of BMDM and SMC from *Apoe^−/−^Nod1^−/−^* and *Apoe^−/−^* mice and exposed them to several proapoptotic stimuli, including UV, the nitric oxide donor GSNO, oxLDL, and the NOD1 ligand, c12-iE-DAP. As shown in Figure 3b, *Nod1* deficiency significantly reduced apoptosis in SMC treated with all four stimuli, as measured by using two different approaches (propidium iodide staining to identify sub-G_0_ cells by flow cytometry and imaging of cleaved caspase-3 immunostaining by confocal microscopy). Similarly, the inactivation of *Nod1* in *Apoe^−/−^* BMDM significantly limited apoptosis induced by UV, GSNO and oxLDL (Figure 3c). c12-iE-DAP, which was able to induce apoptosis in SMC (Figure 3b), failed causing apoptotic cell death in *Apoe^−/−^Nod1^−/−^* and *Apoe^−/−^* BMDM vs. untreated controls.

## 4. Discussion

Atherosclerosis is a complex disease involving lipid accumulation and the central participation of endothelial cells, SMC and monocyte-derived macrophages. The main finding of the present study coincides with our previous observations in human atherosclerosis [9] suggesting a preeminent role for NOD1, a member of the so-called pattern recognition receptors of the innate immunity, not only in endothelial cells, but now also in SMC and macrophages. Although discrete changes in foam-cell formation were found with respect to *Nod1* deficiency in *Apoe^−/−^* mice [27], our results point towards a critical role for NOD1 in features of atherosclerotic plaque stabilization, such as in the intimal collagen content, NC area, FC thickness, leukocyte infiltration and SMC content. Notably, apoptosis of SMC and macrophages lacking *Nod1* is reduced in the lesion area. In addition to this, primary cell cultures of macrophages and SMC from *Apoe^−/−^Nod1^−/−^* mice exposed to different pro-apoptotic stimuli show decreased apoptosis activity. Therefore, our findings demonstrating a direct link between NOD1 and plaque vulnerability in *Apoe^−/−^* mice may be helpful to the clinical practice in terms of the events that precede plaque rupture during atherothrombosis, such as NC formation, the cell-death in the lesion area or the composition of the fibrotic tissue. Moreover, a recent work combining *Nod1* and *Nod2* deficiency under the *Ldlr^−/−^* background shows similar results to those reported in this work [28]; however, in our animal model, whereas *NOD1* was highly upregulated under HFD regime, *NOD2* levels remained unchanged (Figure S9) stressing the relevance of NOD1 in the context of atherosclerosis.

Although we found higher proliferative capacity of cultured *Apoe^−/−^Nod1^−/−^* SMC, this response did not correlate with significant changes in cell proliferation within the atheroma, suggesting that the balance of cell proliferation vs. cell death concerning NOD1 in SMC tilts the balance to reduce cell death and to increase matrix synthesis to stabilize the lesion. Although all cell types within the vessel wall can undergo apoptosis, it is predominantly restricted to macrophages and SMC [29]. In agreement with the well-established proapoptotic function of NOD1 through caspase activity [30,31], our in vivo and in vitro studies reveal a marked reduction in the NC content of apoptotic macrophages and SMC in *Apoe^−/−^Nod1^−/−^* compared to their *Apoe^−/−^* counterparts. It is noteworthy that we found higher anti-apoptotic protection in *Nod1*-deficient neointimal macrophages (3-fold) than in SMC (1.5-fold), which not surprisingly correlates with the greater protection of *Apoe^−/−^Nod1^−/−^* BMDM to foam-cell formation and lipid uptake. Likewise, our in vitro experiments demonstrate that both SMC and macrophages can undergo NOD1-dependent apoptosis not only in response to endoplasmic reticulum stressors (e.g., GSNO, UV) [32,33], but also in response to the novel NOD1 activator, oxLDL [9,34]. Remarkably, although our observations are preliminary, if it is true that SMC respond to the NOD1 classical ligand iE-DAP in terms of cell death, future research on systemic bacterial infection and the dysregulation of the microbioma during vascular remodeling and/or plaque stabilization [35] would be of great interest.

Plaque rupture due to reduced tensile strength of the collagen layer that surrounds the plaque, as well as to endothelial erosion after metabolic or immune insults, are the two possible causes of atherothrombosis. While our previous work already shed light on NOD1 and plaque erosion [9], here we introduce the critical role this receptor may play in plaque rupture. In this sense, the reduced content of mature cross-linked collagen, the large NC, the high inflammation and the thin layer of collagen are synonymous of vulnerable plaques [10]. *Apoe^−/−^* lesions show increased macrophage and neutrophil infiltration followed by a decrease in SMC and collagen content compared to those on *Apoe^−/−^Nod1^−/−^* mice, highlighting important features of plaque vulnerability. Likewise, *Apoe^−/−^* atheromas show, compared to *Apoe^−/−^Nod1^−/−^* lesions, expanded NCs and thin FCs as a consequence of loss of SMC activity, consistent with additional archetypical signs of plaque instability that predict plaque rupture. Remarkably, detailed analysis of the collagen content in both mice models shows a significant difference between thin type III (*Apoe^−/−^*: 6%; *Apoe^−/−^Nod1^−/−^*: 1%) and thick type I (*Apoe^−/−^*: 90%; *Apoe^−/−^Nod1^−/−^*: 95%) fibers. While collagen types I and III are identified in human atherosclerotic plaques, type III collagen has been suggested to be the major platelet activator after plaque rupture evoking ACS [36], further reinforcing the importance of our conclusions in NOD1 on the pathophysiology of atherothrombosis. Nevertheless, uncontrolled collagen accumulation can cause arterial stenosis and changes in fiber composition contribute to the development of arterial stiffness [37,38]. Therefore, future assessment of the dynamic balance between degradation and synthesis of collagen might be required to investigate differential affections by *Nod1* ablation.

Collectively, we dare to suggest that while low leukocyte infiltration in *Nod1^−/−^* endothelium plus reduced apoptosis in *Nod1^−/−^* macrophages may decrease the risk of atherothrombosis, *Nod1^−/−^* SMC balance between higher proliferation rates and decreased apoptotic activity may contribute to thicker FCs with reduced content in pro-thrombotic type III collagen. Despite the limitations in the study of thrombotic events in the classical *Apoe^−/−^* mice model, NOD1 blockade appears to be not only a promising therapeutic strategy to prevent atherothrombosis without compromising host defense, but also a useful biomarker for earlier detection and subsequent treatment of patients with subclinical silent vulnerable lesions before resulting in ACS. Additionally, the beneficial effects of this treatment might be extended to other forms of arterial remodeling, such as aneurysm formation and restenosis after angioplasty, as well as chronic inflammatory diseases associated with defective macrophage apoptosis including Crohn’s disease and chronic obstructive pulmonary disease.

## Supplementary Materials

The following are available online at https://www.mdpi.com/2073-4409/9/9/2067/s1, Figure S1: HFD-induced body weight increase and lipid profile changes in *Apoe^-/-^* mice are not mediated by *Nod1*. Figure S2: Inset of NOD1 from atherogenic human coronary arteries (from Figure 1c) and NOD1 abundance in consecutive sections of human atherosclerotic coronary arteries presenting cholesterol crystals. Figure S3: Quantification of the immunoblots shown in Figure 1d. Figure S4: mRNA levels of scavenger receptors in athero-prone *Apoe^-/-^* mice in the presence and absence of *Nod1*. Figure S5: *Nod1* does not affect lipid atherosclerotic area in *Apoe^-/-^* mice. Figure S6: *Apoe^-/-^Nod1^-/-^* mice present less leukocyte cell content in blood comparing to their *Apoe^-/-^* counterparts. Figure S7: *Nod1* inactivation in mice results in a lower number of total CD45^+^ cells and neutrophils in spleen, while there are no significant changes in other white cells subsets. Figure S8: *Nod1* inactivation in cultured SMCs promotes faster cell cycle entry into S-phase. Figure S9: mRNA levels of Nod2 in the aortic arch of athero-prone *Apoe^-/-^* mice in the presence and absence of *Nod1*.

## Abbreviations

**Abbreviations**	**Acronyms**
ACS	Acute coronary syndrome
BMDM	Bone-marrow derived macrophage
FC	Fibrous cap
HE	Haematoxylin-eosin
HFD	High-fat diet
iE-DAP	γ-D-glutamyl-meso-diaminopimelic acid
NC	Necrotic core
ORO	Oil Red O
oxLDL	Oxidized low-density lipoprotein
SMC	Smooth muscle cell
