# Supplementary material for: Deletion or Inhibition of NOD1 Favors Plaque Stability and Attenuates Atherothrombosis in Advanced Atherogenesis
Source: Cells. 2020 Sep 10;9(9):2067. doi: 10.3390/cells9092067 (PMC7564689; doi:10.3390/cells9092067)
Supplement: Supplementary file 1 [file cells-09-02067-s001.pdf]

**SUPPLEMENTAL MATERIAL**

# Deletion or inhibition of NOD1 favours plaque stability and attenuates atherothrombosis in advanced atherogenesis

Silvia González-Ramos[1,2,*,$], PhD; Victoria Fernández-García[1,2,$], MSc; Miriam Recalde[1], MSc; Cristina Rodríguez[2,3], PhD; José Martínez-González[2,4], PhD; Vicente Andrés[2,5], PhD; Paloma Martín-Sanz[1,2], PhD; Lisardo Boscá[1,2,*], PhD

*[1]Instituto de Investigaciones Biomédicas Alberto Sols (CSIC-UAM), Madrid, Spain;*
*[2]Centro de Investigación Biomédica en Red en Enfermedades Cardiovasculares (CIBERCV), Madrid, Spain;*
*[3]Institut de Recerca del Hospital de la Santa Creu i Sant Pau-Programa ICCC, IIB Sant Pau, Barcelona, Spain;*
*[4]Instituto de Investigaciones Biomédicas de Barcelona (CSIC-IIBB), IIB Sant Pau, Barcelona, Spain;*
*[5]Centro Nacional de Investigaciones Cardiovasculares (CNIC), Madrid, Spain.*

**Running title:** NOD1 deficiency promotes atheroma stability

**Address correspondence to:**
Lisardo Boscá *or* Silvia González-Ramos
Instituto de Investigaciones Biomédicas Alberto Sols
C/Arturo Duperier, 4
28029 Madrid
Phone +34-(0)91-497-2747 *or* -5345
Email lbsoca@iib.uam.es or sgramos@iib.uam.es

**Supplementary Figures**

***a***

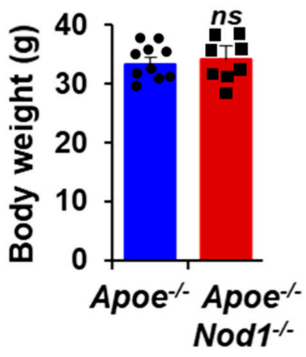


***b***

| mg/dL | *Apoe*$^{-/-}$ | *Apoe*$^{-/-}$*Nod1*$^{-/-}$ |
|---|---|---|
| **Triglycerides** | 118.1 ± 2.3 | 112 ± 4.2 |
| **LDL** | 237.1 ± 4.6 | 244.6 ± 6.4 |
| **HDL** | 101.1 ± 4.5 | 103.6 ± 4.4 |
| **Total Cho.** | 372.1 ± 3.4 | 382.6 ± 8.1 |
| **Free Cho.** | 267.6 ± 6.7 | 279.9 ± 8.8 |

**Figure S1. HFD-induced body weight increase and lipid profile changes in *Apoe*$^{-/-}$ mice are not mediated by *Nod1*. (a)** Body weight in *Apoe*$^{-/-}$ (*n*=10) and *Apoe*$^{-/-}$*Nod1*$^{-/-}$ (*n*=8) mice after 16 weeks on a HFD. **(b)** Serum concentration of triglyceride (TGA), high-density lipoproteins (HDL), low-density lipoproteins (LDL), total cholesterol (TCHO) and free cholesterol (FCHO) in the same cohort of mice. Data is represented as mean ± s.e.m. of the indicated number (*n*) of repeats. ***ns***; Non-significant by Student's *t* test.

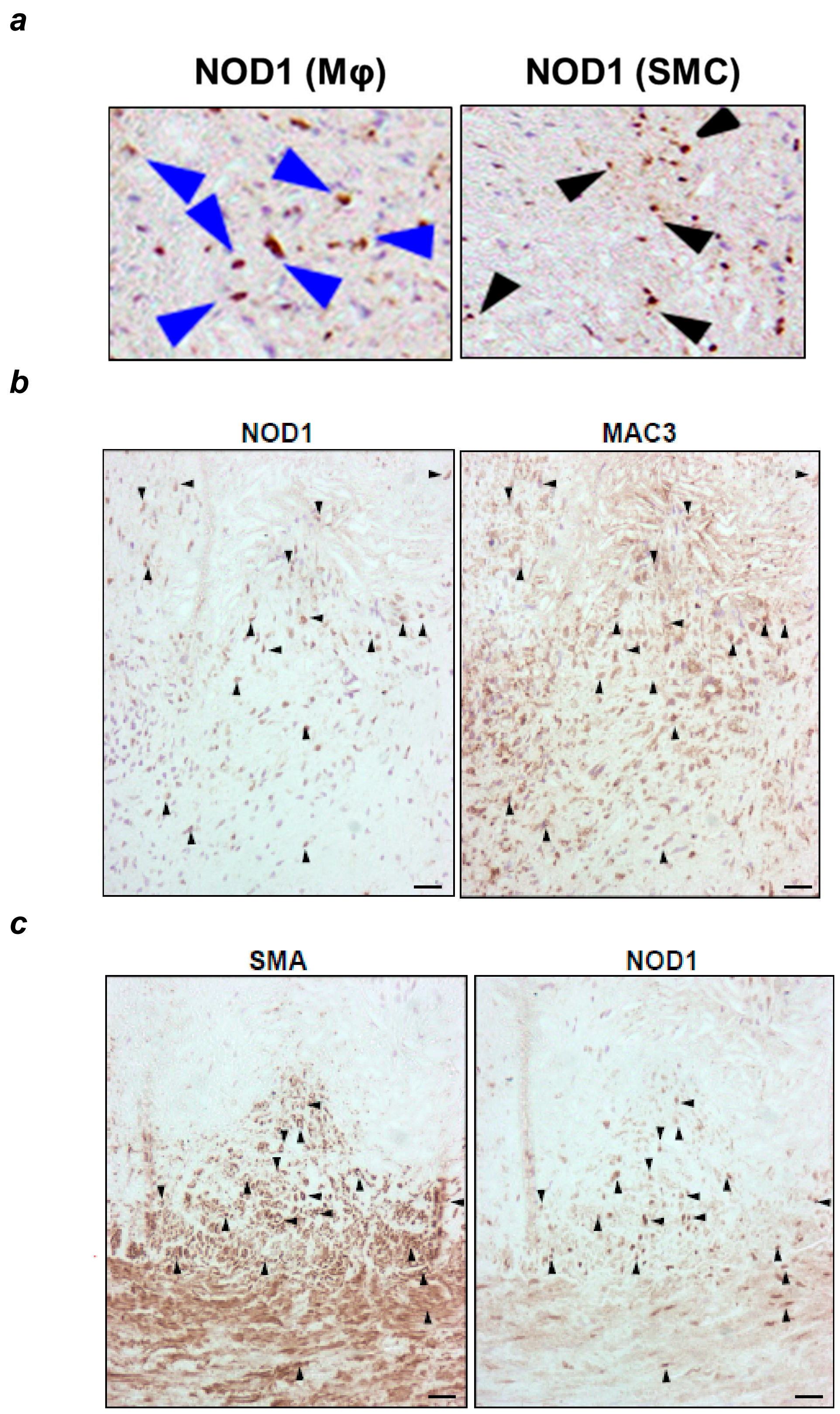


**Figure S2. Inset of NOD1 from atherogenic human coronary arteries (from Fig. 1c) and NOD1 abundance in consecutive sections of human atherosclerotic coronary arteries presenting cholesterol crystals. (a)** Amplification of atherogenic sections from Fig. 1c. **(b)** NOD1 co-localises with macrophage-rich areas (Mac3$^{+}$-cells) in the media and intima of atherosclerotic human arteries. **(c)** Representative images showing the immunohistochemical analysis of SMA and NOD1.Blue arrows point to NOD1$^{+}$ cells in macrophages; black arrows point out NOD1$^{+}$ cells in the smooth muscle cells.

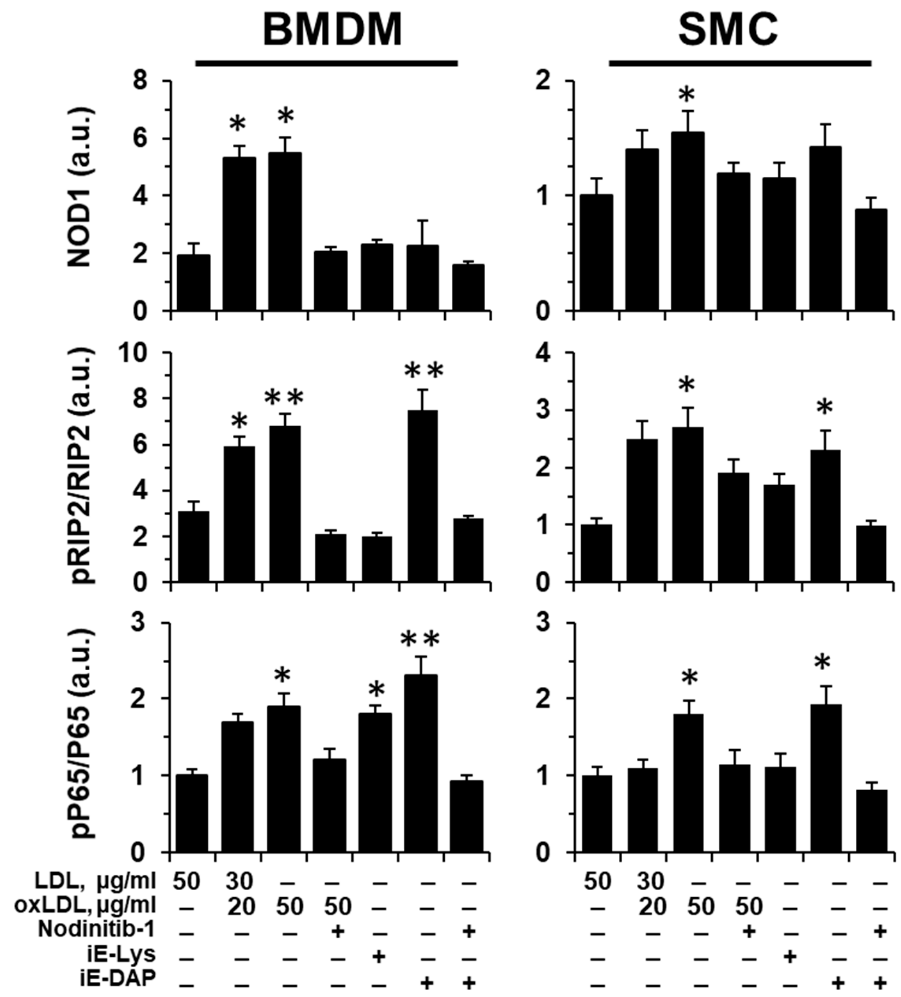


**Figure S3. Quantification of the immunoblots shown in Fig. 1d.** Analysis of NOD1, *p*RIP2/RIP2, pP65/P65 in *Wt* BMDM (n=6) and SMC (n=4) pre-treated with the NOD1 inhibitor Nodinitib-1 and/or stimulated with native LDL (as control for lipid load in the medium), oxLDL, c12-iE-DAP (an agonist for NOD1) and iE-Lys (an iE-DAP analogue that fails to activate NOD1) and c12-iE-DAP (an agonist for NOD1) for 24h or 48h, respectively. Protein levels were normalized to tubulin. Data are represented as mean ± s.e.m. of the indicated number (*n*) of independent experiments. **P* < 0.05, ***P* < 0.01 *vs.* the LDL condition by Student's *t* test. a.u., arbitrary units.

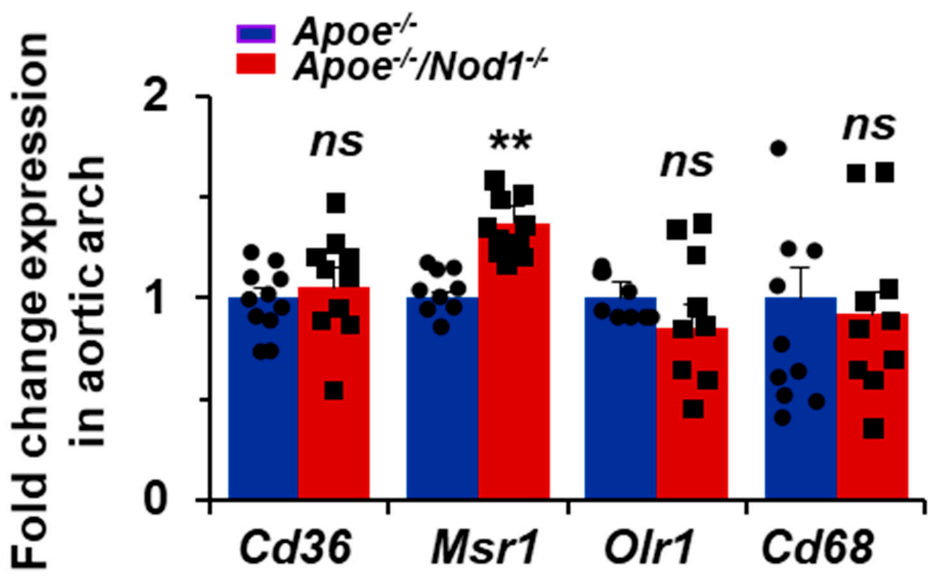


**Figure S4. mRNA levels of scavenger receptors in athero-prone *Apoe*$^{-/-}$ mice in the presence and absence of *Nod1*.** Quantitative PCR analysis of the scavenger receptors *Cd36,* mouse scavenger receptor 1 (*Msr1*), oxidised low-density receptor 1 (*Olr1)* and the low-density lipoprotein receptor *Cd68* expression in the aortic arch of *Apoe*$^{-/-}$ (*n*=10) and *Apoe*$^{-/-}$*Nod1*$^{-/-}$ (*n*=10) mice after 16 weeks on HFD. *m*RNA levels were normalised to *mRplp0* expression. Data are represented as mean ± s.e.m. of the indicated number (*n*) of repeats. ***P* < 0.01 *vs. Apoe*$^{-/-}$ by Student's *t* test. ***ns***; Non-significant by Student's *t* test.

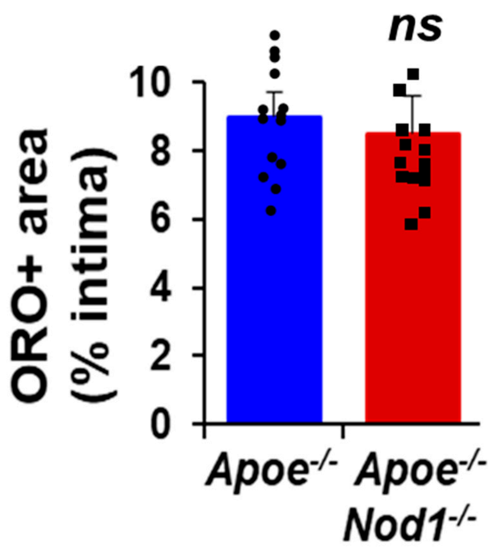


**Figure S5. Nod1 does not affect lipid atherosclerotic area in *Apoe*$^{-/-}$ mice.** Quantification of positive Oil Red O (ORO) lesion area in the semilunar valve cups of *Apoe*$^{-/-}$ (*n*=13) and *Apoe*$^{-/-}$*Nod1*$^{-/-}$ (*n*=13) mice fed HFD for 16 weeks. Data are represented as mean ± s.e.m. of the indicated number (*n*) of repeats. Non-significant by Mann-Whitney U test. ***ns***; Non-significant by Student's *t* test.

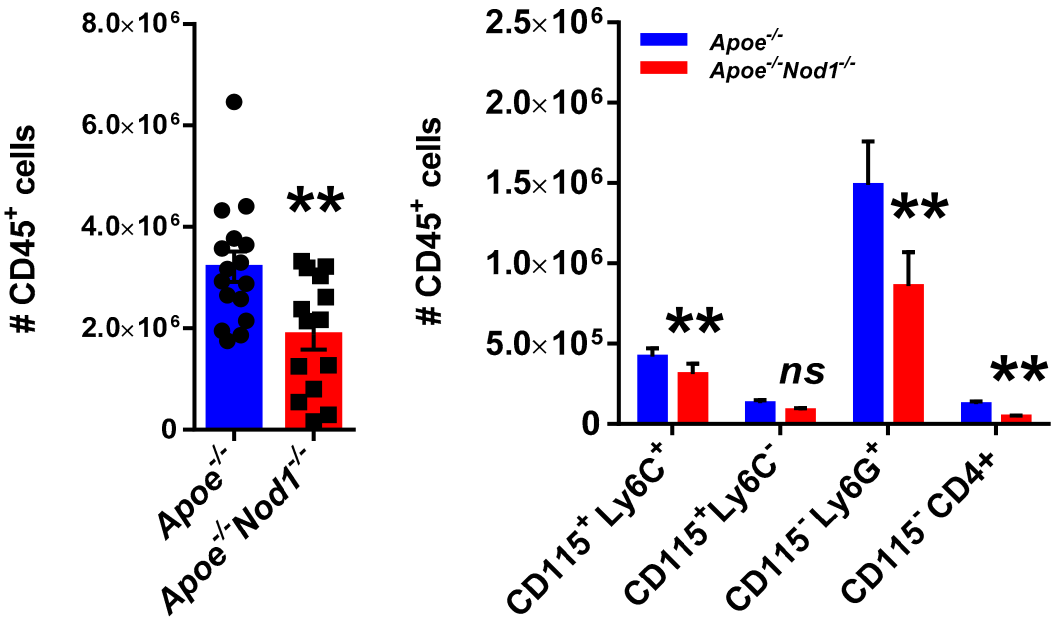


**Figure S6. *Apoe$^{-/-}$Nod1$^{-/-}$* mice present less leukocyte cell content in blood comparing to their *Apoe$^{-/-}$* counterparts.** Quantification of leukocyte, monocyte/macrophage, neutrophil and CD4+ lymphocyte cells in blood collected from *Apoe$^{-/-}$* (*n*=16) and *Apoe$^{-/-}$Nod1$^{-/-}$* (*n*=16) mice (16 weeks HFD-fed), performed by flow cytometry. Data are represented as mean ± s.e.m. of the indicated number (*n*) of repeats. **P* < 0.05, ***P* < 0.01 *vs. Apoe$^{-/-}$* by Student's *t* test. ***ns***; Non-significant by Student's *t* test.

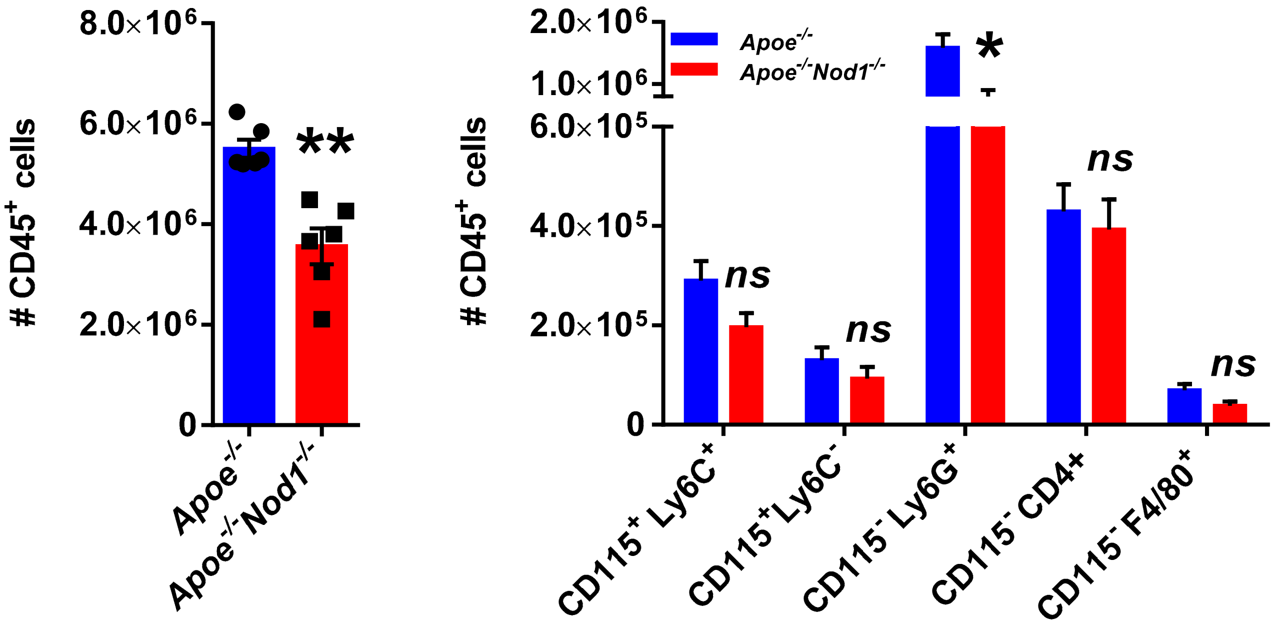


**Figure S7. *Nod1* inactivation in mice results in a lower number of total CD45$^{+}$ cells and neutrophils in spleen, while there are no significant changes in other white cells subsets.** Leukocyte, monocyte/macrophage, neutrophil and CD4$^{+}$ lymphocyte number of cells was assessed in the spleen obtained from *Apoe$^{-/-}$* (*n*=6) and *Apoe$^{-/-}$Nod1$^{-/-}$* (*n*=6) mice (16 weeks HFD-fed), using flow cytometry techniques. Data are represented as mean ± s.e.m. of the indicated number (*n*) of repeats. **P* < 0.05, ***P* < 0.01 *vs. Apoe$^{-/-}$* by Student's *t* test. ***ns***; Non-significant by Student's *t* test.

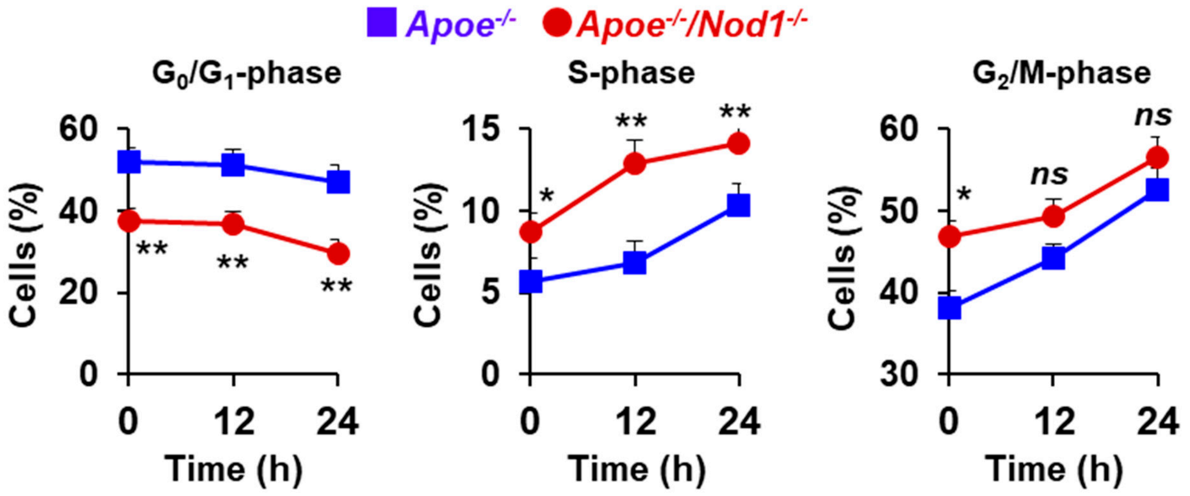


**Figure S8. *Nod1* inactivation in cultured SMCs promotes faster cell cycle entry into S-phase.** Cell cycle profile of primary vascular SMCs from *Apoe*$^{-/-}$ (*n*=10) and *Apoe*$^{-/-}$*Nod1*$^{-/-}$ (*n*=10) mice was assessed in a flow cytometer by propidium iodide staining. Cells were synchronised in $G_0/G_1$ phase by 72-h serum deprivation and restimulated for the indicated times. Results are average representative of three independent experiments. Data are represented as mean ± s.e.m. of the indicated number (*n*) of repeats. **P* < 0.05, ***P* < 0.01 *vs. Apoe*$^{-/-}$ by Student's *t* test. ***ns***; Non-significant by Student's *t* test.

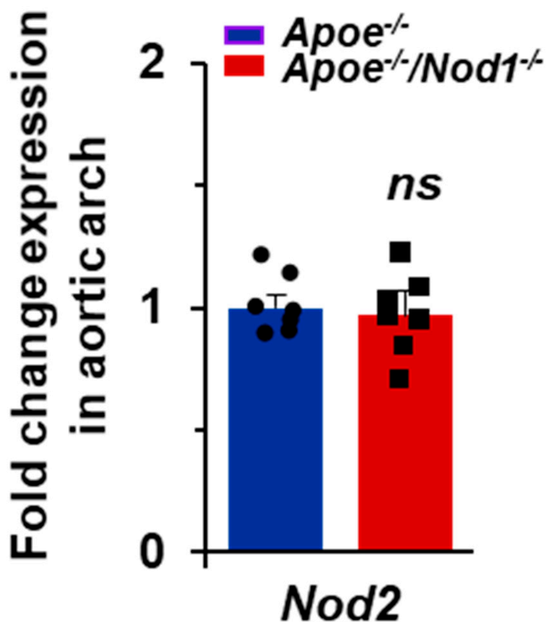


**Figure S9. mRNA levels of Nod2 in the aortic arch of athero-prone *Apoe*$^{-/-}$ mice in the presence and absence of *Nod1*.** Quantitative PCR analysis of *Nod2* expression in the aortic arch of *Apoe*$^{-/-}$ (*n*=7) and *Apoe*$^{-/-}$*Nod1*$^{-/-}$ (*n*=7) mice after 16 weeks on HFD. *m*RNA levels were normalised to *mRplp0* expression. Data are represented as mean ± s.e.m. of the indicated number (*n*) of repeats. ***ns***; Non-significant by Student's *t* test.
